# Cardiac Magnetic Resonance in Heart Failure: Diagnostic and Prognostic Assessments

**DOI:** 10.3390/jcdd12060200

**Published:** 2025-05-23

**Authors:** Sara Pezzini, Francesca Daus, Giorgia Galli, Andrea Farina, Gabriele Fragasso, Roberto Spoladore

**Affiliations:** 1 Health Science Department, University of Milan-Bicocca, 20126 Milan, Italy; s.pezzini1@campus.unimib.it (S.P.); f.daus@campus.unimib.it (F.D.); 2 Cardiovascular Imaging Unit, Division of Cardiology, Alessandro Manzoni Hospital, 23900 Lecco, Italy; g.galli@asst-lecco.it; 3 Heart Failure Clinic, Division of Cardiology, Alessandro Manzoni Hospital, 23900 Lecco, Italy; a.farina@asst-lecco.it; 4 Heart Failure Unit, Division of Cardiology, IRCCS Vita-Salute San Raffaele University Hospital, 20132 Milan, Italy; gabriele.fragasso@hsr.it

**Keywords:** cardiac magnetic resonance, heart failure, diagnosis, prognosis

## Abstract

With its high spatial resolution and tissue contrast, cardiac magnetic resonance (CMR) is an extremely flexible imaging technique that enables a thorough morphological and functional evaluation of the heart and vascular system. From diagnosis to treatment planning and risk assessment, CMR is being utilized more and more in the evaluation of patients with heart failure (HF). CMR offers a variety of techniques for characterizing myocardial tissue, aiding in identifying the cause of HF, as well as whole-heart cine imaging for precise measurement of biventricular dimensions and function. The aim of this review is to the describe the role of CMR in chronic heart failure, in particular for diagnostic workups, differential diagnosis, and information on how CMR influences treatment planning choices.

## 1. Introduction

Cardiac magnetic resonance (CMR) is the gold standard for evaluating the volumes and function of the heart. In addition to being extremely reproducible and independent of geometric assumptions, it has been demonstrated to be superior to both two- and three-dimensional (2D) echocardiography [1]. The 2021 European Society of Cardiology (ESC) guidelines for the diagnosis and treatment of acute and chronic heart failure state that CMR is the preferred imaging modality for patients with complex congenital heart disease and the recommended alternative test to echocardiography for evaluating biventricular volumes and function in patients with suboptimal or poor acoustic windows (such as patients with obesity or pulmonary disease) [2]. The presence and severity of myocardial fibrosis, myocardial edema, infiltration, and iron overload can be determined by the non-invasive cardiac tissue characterization capabilities of CMR. The assessment of functional tissue abnormalities helps in the differential diagnosis of heart disease, contributing to guidance for customized management and treatment [2]. According to the 2021 ESC Guidelines, CMR should be used to differentiate between ischemic and non-ischemic causes of dilated cardiomyopathy (DCM), particularly when imaging or clinical findings are unclear. With a Class I recommendation, CMR is acknowledged for its involvement in identifying the etiology of heart failure in a variety of cardiomyopathic diseases, including Fabry disease, amyloidosis, sarcoidosis, and myocarditis. The differential diagnosis of restrictive cardiomyopathies with comparable characteristics, like left ventricular (LV) hypertrophy, is made easier with the additional use of parametric mapping. Lastly, in order to guide revascularization decisions for patients with heart failure and coronary artery disease (CAD), CMR stress imaging is recommended for the evaluation of myocardial ischemia and viability [2].

## 2. CMR Protocols

The magnetic characteristics of atomic nuclei (in this case, the hydrogen atom) represent the fundamental principle of magnetic resonance imaging (MRI). A sequence of radiofrequency pulses, magnetic gradient field switches, and timed data captures are used to expose tissue to a strong external magnetic field and create an image that depicts the tissue’s magnetic characteristics in the imaging field. The order, timing, size, and form of the radiofrequency pulses can be changed to create images that highlight various tissue features [3]. The most prevalent tissue characteristics are T1 and T2 constants, which generally reveal high signals from fat in T1-weighted images and high signals in myocardial edema or inflammatory regions in T2-weighted images. Furthermore, MRI contrast dyes can be employed to identify cardiac fibrosis or infiltration and to further enhance certain tissue features [3]. Anatomical imaging and tissue characterization are generally evaluated by “spin echo sequences.” Cardiac function imaging is performed using faster sequences, such as gradient echo (GRE) and steady-state free precession (SSFP). Usually, blood appears dark on spin echo imaging while it is bright on GRE sequences.

### 2.1. Assessment of Left and Right Ventricular Function

Contiguous stacks of cine images covering the entire heart in either the axial plane or the LV short axis are typically used for CMR assessments of the LV and right ventricular (RV) volumes, mass, and ejection fraction (EF). Data are gathered during several cardiac cycle phases (usually at least 30) and then reconstructed into motion picture images [4]. The blood–myocardium interface is well defined by SSFP sequences, which also allow precise identification of wall motion abnormalities. The Simpson’s approach of multiple disc-derived measurements is used to generate volumetric and functional indices, such as biventricular end-diastolic and end-systolic volumes, EF, stroke volume, and LV mass [4].

### 2.2. Diastolic Function

Heart failure with preserved systolic ejection fraction accounts for nearly 50% of all HF patients. Diastolic function can be evaluated by CMR in different ways. Like echocardiography, flow velocity-encoded CMR may quantify the E/A ratio of the mitral inflow as well as early diastolic (E) and atrial systolic (A) peak flow velocities. An additional post-processing technique of CMR, useful in the assessment of diastolic function, is CMR feature tracking (CMR-FT). It is based on pattern matching methods on common cine pictures, providing longitudinal, radial, and circumferential strain measurements [5].

### 2.3. Tissue Characterization

Several features of cardiac tissue can be examined using a variety of CMR techniques. The most widely used techniques include parametric T1, T2, and T2* mapping, T1- weighted and T2-weighted imaging, and late gadolinium enhancement (LGE) after contrast delivery.

#### 2.3.1. T1-Weighted Imaging, and T1 and ECV Mapping

The T1 relaxation time is a constant that depends on the tissue composition and the MRI scanner’s field strength. The myocardium shows an intermediate signal in T1-weighted images, while fat, which has a short T1, appears bright. T1 mapping creates a pixel-wise map with the tissue’s T1 relaxation time expressed as absolute values, which can then be shown using threshold scales or color to enable quantitative visual perception. The most popular technique for T1 mapping is the modified look-locker inversion recovery (MOLLI) pulse sequence that creates T1 relaxation curves by making a number of acquisitions over consecutive heartbeats. The primary physiologic factors that contribute to elevated native myocardial T1 comprise an increase in interstitial space due to edema (for instance, in cases of acute infarction or inflammation), fibrosis, and inflammatory processes, including amyloid buildup. Water–protein interactions and the amount of fat or iron in the myocardium might lower native T1 values (e.g., in Fabry disease) [4,6]. Since gadolinium-based contrast agents are extracellular and extravascular, post-contrast T1 values are lower in tissues where the extracellular space has expanded. Native and post-contrast T1 mapping can be used to quantify the extracellular volume (ECV) of tissue. The ECV of the normal myocardium has been reported to be between 24 and 28% [7]. ECV expansion has been shown in a variety of cardiac conditions and is most commonly caused by excessive collagen deposition or invasive processes (fat, protein), making it a measure of myocardial fibrosis or, more broadly, pathologically expanded interstitial space. Low ECV values are found in lipomatous metaplasia and fat [8].

#### 2.3.2. T2-Weighted Imaging and T2 Mapping

T2-weighted imaging can be used in qualitative or semi-quantitative detection of myocardial edema and inflammation, which prolong T2 relaxation times and make edematous tissues appear brighter than normal remote myocardium. Standard T2-weighted imaging of myocardial edema typically uses turbo spin echo (TSE), with or without fat saturation pulses, mostly in conjunction with dark blood preparations [4,6,9]. The most commonly used sequence is the short T1 triple inversion recovery (STIR) fast spin echo sequence, which suppresses signals from fat and blood to improve the contrast between edematous tissue, the normal myocardium, and the LV cavity. A T2 mapping sequence measures the T2 transverse relaxation time in each voxel and then produces a parametric image where the intensity reflects the measured T2 value. The T2 values can be visually represented on color scales and then quantified further within areas of interest [4,6,9].

#### 2.3.3. T2* Sequence

Iron deposition increases the relaxation parameter T2*, which is mostly caused by local magnetic field heterogeneity. T2* mapping allows for the quantification of tissue iron concentration by measuring the T2* relaxation of cardiac tissue from GRE sequences [4,6].

#### 2.3.4. Early and Late Gadolinium Enhancement (LGE)

Additional tissue characterization is possible with post-contrast T1-weighted imaging. Immediately following a gadolinium-based contrast agent injection, areas that are severely hypovascular will not improve. In acute myocardial ischemia, early gadolinium-enhanced imaging is usually carried out a few minutes after contrast is administered in order to identify thrombus or microvascular blockage. An equilibrium between contrast uptake, distribution, and washout develops with prolonged contrast injection delays. Collagen-associated extracellular water has a larger volume of distribution and slower contrast kinetics in areas of infarction or localized fibrosis. LGE CMR, which produces high-resolution pictures of scar or fibrosis, is typically obtained ten minutes following contrast injection using T1-sensitive inversion recovery techniques. Typically with LGE, regions of myocardial damage or fibrosis look bright, whereas the signal from the normal myocardium is “nulled” or black [4,6].

LGE images can be evaluated numerically or visually by comparing their relative augmentation to the background. LGE is often of prognostic significance and can be diagnostic of the underlying etiology; its pattern and degree vary depending on the underlying illness process.

## 3. CMR Applications for Differential Diagnosis and Prognosis in Heart Failure

As a substantial body of evidence has demonstrated, CMR has become a pivotal diagnostic and prognostic instrument for a range of cardiomyopathies, all of which have the potential to result in heart failure.

### 3.1. Ischemic and Non-Ischemic Dilated Cardiomyopathy (DCM)

While it is a widely accepted fact that ischemic cardiomyopathy is the most prevalent form of cardiovascular disease [10], it is also known that dilated cardiomyopathy (DCM) encompasses a broad spectrum of non-ischemic conditions, ultimately leading to heart failure. As the pathophysiology underlying each group differs, it is crucial to make an accurate distinction between them for the purpose of prognosis and therapeutic management of patients. Cardiac magnetic resonance (CMR) in the setting of DCM allows a reproducible assessment of the left ventricular ejection fraction (LVEF) and ventricular volumes, as well as tissue characterization through gadolinium contrast, parametric mapping, and feature tracking [11]. Tissue characterization has an established role in defining the etiology of various forms of dilated cardiomyopathy, allowing an etiology-oriented risk stratification and treatment. The myocardial fibrosis pattern (i.e., LGE) is the most useful tool in differential diagnosis [11]. For example, patchy midwall LGE may suggest post-myocarditis or dystrophinopathy, and fatty replacement within the left ventricular wall may suggest left-dominant arrhythmogenic right ventricular cardiomyopathy [1]. As demonstrated in a number of previous studies [12,13], the ischemic LGE pattern is consistent with the pathophysiology of coronary occlusion. Indeed, approximately 15 min after the onset of an ischemic event, myocite necrosis spreads from the subendocardium to the subepicardium. Therefore, the typical ischemic pattern always involves the subendocardium and may extend to the subepicardium (thereby resulting in a transmural pattern) and is consistent with coronary distribution [14]. Myocardial viability assessment has a crucial role in this context, as it can guide clinical decision making on revascularization. It mainly relies on the visual estimation of scar thickness extent (i.e., LGE) in comparison to the total wall thickness. A universal cut-off still has to be clearly defined, even though an LGE extension that exceeds 50% of the total wall thickness is usually considered a contraindication to revascularization. A correlation between viable myocardium detected at CMR and improved outcomes after revascularization was shown by Gerber et al. [15].

Recently, a viability sub-study of the STICH trial cast doubt on this concept since it showed no link between viability and benefit from CABG [16]. However, this finding may not be widely applicable to the general population, as it was a non-randomized study that included a minority of patients with non-viable myocardium. In addition, the imaging methods employed, i.e., SPECT and dobutamine-stress echocardiography (DSE), were not able to provide a direct assessment of the myocardial scar and its transmurality.

Non-ischemic LGE patterns are not as clearly defined and are detected in fewer than 50% of DCM patients. Myocardial fibrosis is due to collagen accumulation and is a well-known substrate for ventricular arrhythmia [17]. A recent meta-analysis involving a sample of more than 7700 patients highlighted its ability to predict multiple adverse outcomes, including all-cause mortality and arrhythmic events [18]. The septum was the most commonly affected segment, while the midwall pattern was the most prevalent. Both of these features emerged as risk predictors of adverse outcomes in this context. However, free wall involvement has also been observed, as well as multiple patterns, including subepicardial and transmural. The underlying pathophysiology is yet to be fully elucidated. Another meta-analysis combining 2948 patients with DCM demonstrated an association between fibrosis detected by LGE and SCD, with an arrhythmic endpoint that remained significant among patients with LVEF > 35% [19]. Also, the increase in extracellular volume (ECV) assessed by T1 mapping reflected diffuse fibrosis not detectable with LGE, and it was associated with a higher risk of all-cause mortality in a prospective observational study involving 637 patients with DCM [20]. Despite these data, there is no conclusive evidence to demonstrate that patients with severe LV systolic dysfunction and without fibrosis are not at risk of SCD, and no fibrosis threshold has been identified beyond which the arrhythmic risk is considered significantly increased. Based on these findings, the presence of fibrosis on CMR is a recognized risk factor for SCD and all-cause mortality in DCM, but the mere presence of fibrosis is not a sufficient criterion for ICD implantation in these patients [21].

CMR is also emerging as an irreplaceable tool in identifying genotype–phenotype correlations in gene-positive DCM. A recent Spanish cohort study involving 600 genotyped patients with DCM observed that different LGE patterns were related to underlying genetic variants. In particular, subepicardial LGE was more frequently associated with Dystrophin (*DMD*), Desmoplakin (*DSP*), and Filamin C (*FLNC*) variants, while it was rare or absent with troponin T type 2 (*TNNT2*), RNA Binding Motif Protein 20 (*RBM20*), and Myosin Heavy Chain 7 (*MYH7*) variants. Unspecific patterns were observed with Titin (*TTN*), BCL2 associated Athanogen 3 (*BAG3*), Lamin A/C (*LMNA*), and Myosin Binding Protein C (*MYBPC3*) mutations.

**Figure 1:**
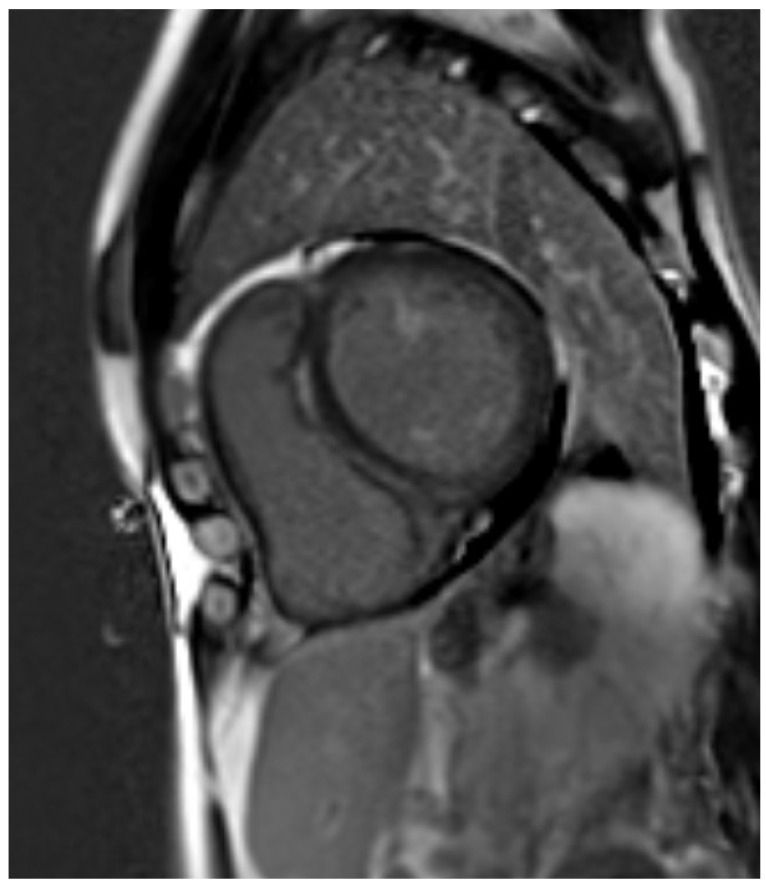
Dilated cardiomyopathy (LMNA mutation) with diffuse LGE showing «ring-like» pattern.

Moreover, together with LGE extent, LGE patterns could predict end-stage heart failure and major ventricular arrhythmias, thus providing useful information for primary prevention ICD implantation [22,23]. LGE presence and septal location were also found to be predictors of major arrhythmic events in a recent multicenter cohort study involving DCM patients [24]. A notable interesting LGE location was the ring-like pattern (i.e., > 3 or more adjacent segments using the AHA 17 segments model) (Figure 1). Although uncommon, it was more frequently found in DCM patients with underlying genetic mutations (specifically, *FLNC* and *DSP*) and was clearly associated with a significantly high risk of adverse outcomes, including life-threatening arrhythmias, all-cause death, and heart transplantation [25]. Its predictive value was shown to be independent of total LGE burden and additional risk factors, thereby highlighting the necessity for closer follow up and thorough evaluation for ICD placement [26].

### 3.2. Acute Myocarditis

**Figure 2:**
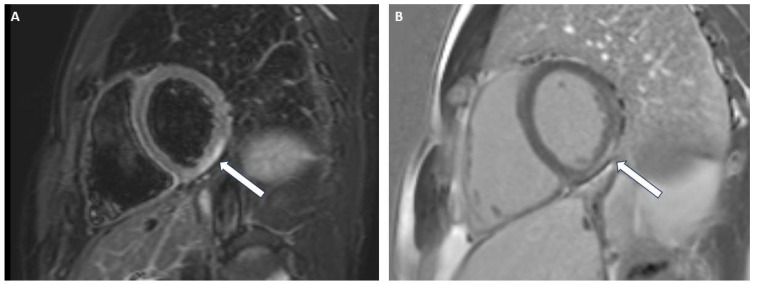
Short axis views of left ventricular acute myocarditis. Myocardial aedema in T2 weighted sequence (**A**); subepicardial LGE (**B**).

CMR has also become pivotal in the non-invasive assessment of clinically suspected myocarditis owing to its capacity to detect myocardial inflammation. Its high diagnostic accuracy permitted the identification of Lake Louise criteria, largely used in clinical and research settings and supported by a large body of evidence. The 2018 update drew attention to the increasing importance of T1 and T2 mapping, as it included these newer techniques in addition to T2W images and LGE [27]. Elevated T2 mapping values are indicative of active inflammation within the myocardium and appear to be more precise than T2W imaging and T1 mapping in this setting, as the latter may also be increased in chronic conditions (Figure 2). Furthermore, T2W images are frequently suboptimal due to technical limitations [28]. As signs of acute inflammation decrease quickly over time, CMR offers optimal diagnostic performance in the first few weeks after clinical onset.

Acute myocarditis can present in a variety of ways, ranging from infarct-like presentation to severe heart failure leading to cardiogenic shock. Despite the fact that endomyocardial biopsy (EMB) remains the gold standard examination for the diagnosis of myocarditis in high-risk cases, CMR has become increasingly important as a non-invasive method to confirm diagnosis and provide risk stratification. In addition, its accuracy in detecting even slight tissue abnormalities allows for effective distinction between mild and severe cases of myocarditis (i.e., acute fulminant myocarditis). In the latter case, a marked increase in wall thickness, biventricular function impairment, extensive edema, and LGE suggest a more aggressive course of the disease, warranting prompt treatment. Following resolution of the acute process, a significant number of patients will transition to heart failure as a result of incomplete left ventricular recovery. Several CMR features have recently been shown to predict outcomes in this context. The largest body of evidence indicates that LGE is the most effective risk predictor of all-cause mortality, cardiac mortality, and major adverse cardiac events (MACE), with its extent and location (in particular, antero-septal) being as important as its mere presence [29,30,31]. In addition, poorer outcomes have been identified in patients with no signs of edema (i.e., negative STIR or T2 mapping), as this may indicate irreversible injury with no possibility of myocardial recovery [32,33]. Strain analysis showed interesting results as well, as multiple studies found a significant correlation between lower initial left ventricular and atrial strains and adverse outcomes in patients with myocarditis [34].

Moreover, LGE patterns and extent provide further information about the pathological process, as, if persistent, it relates to permanent myocardial injury once the acute phase has resolved (Figure 2). Higher prevalence of a subepicardial pattern and inferolateral wall involvement has been reported, although not exclusively. It has also been shown to have a prognostic role in predicting adverse outcomes in this setting [27,28].

### 3.3. Chemotherapy-Induced Cardiotoxicity

Cardiotoxicity is a well-known side effect of various oncological therapies. Cancer therapeutics–related cardiac dysfunction (CTRCD) has recently been defined as a decrease in the LVEF of >10 percentage points, to a value of <35%, confirmed in repeated exams [35]. Identifying this condition may often be challenging, as the patient may remain asymptomatic until advanced stages of the disease, while the onset of the condition itself may occur years after the administration of oncological therapy. In this context, echocardiography is the most frequently employed tool, although CMR has some advantages over it. Alongside enhanced precision in assessing biventricular morphology and function, CMR may also detect subtle tissue changes preceding the loss of function. A number of CMR techniques (i.e., myocardial tagging, feature tracking, and fast strain-encoded) are widely accepted and validated in the assessment of myocardial strain, which, as is well established, can predict subclinical LV disfunction prior to EF deterioration [36,37]. Moreover, cardiotoxicity often presents as an inflammatory condition marked by edema, increased extracellular volume (ECV), and diffuse interstitial fibrosis. As previously stated, various CMR techniques (i.e., T1 native and post-contrast mapping, T2W images, T2 mapping) are able to detect inflammation-related tissue changes. As demonstrated by Neilan et al. [38], there is a strong correlation between antracycline-based treatment and ECV elevation, leading to diastolic disfunction and increased left atrial volume. A recent study yielded similar findings [39], as it demonstrated that native T1 and ECV levels remained elevated years after the administration of antracycline treatment. It is yet to be determined whether these findings can provide guidance for the planning of oncological treatment. As focal fibrosis was an uncommon finding in this subset of patients, LGE was usually a less sensitive marker in this setting [40]. Furthermore, CMR is an invaluable tool when it comes to assessing RT-induced cardiotoxicity, given its ability to examine pericardium and valve morphologies. Indeed, it is widely acknowledged that RT can trigger acute pericarditis, which can potentially result in constrictive pericarditis. Concurrently, RT-induced valve disease may present with thickening, calcifications, or fibrosis of the apparatus, mainly associated with significant regurgitation [41].

### 3.4. Hypertrophic Cardiomyopathy (HCM)

**Figure 3:**
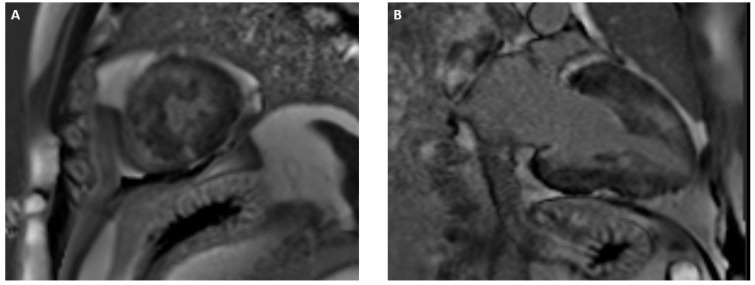
Short axis (**A**) and long axis (**B**) views of left ventricular LGE patchy distribution in sarcomeric hypertrophic cardiomyopathy.

The key feature of HCM is the hypertrophy and non-dilation of LV, which cannot be explained by abnormal loading conditions or other cardiac, metabolic, or systemic diseases [42]. CMR is widely recognized as an essential tool for evaluating and monitoring patients with HCM, as well as for enabling differential diagnoses with phenocopies. In comparison with echocardiography, it provides enhanced precision in septal wall thickness measurements. This is due to the fact that right ventricle structures, which can potentially hinder this measurement (i.e., moderate band and crest supraventricularis), are more clearly defined. In addition, its superior spatial and temporal resolutions enable the effective detection of HCM-associated structural abnormalities, including myocardial crypts, apical displacement of papillary muscles, mitral valve abnormalities, aneurisms, and apical thrombi [43,44,45]. The phenotype of hypertrophic cardiomyopathy (HCM) explains why arrhythmic risk stratification should not rely solely on the assessment of LVEF, which is typically reduced only in more advanced stages of the disease. In patients with HCM, the annual risk of SCD or appropriate ICD interventions is estimated to be approximately 0.8% [46]. Several strategies have been proposed to identify high-risk patients who may be candidates for primary prevention ICD implantation. The ESC guidelines recommend using the HCM Risk-SCD calculator [47] to estimate the 5-year risk of SCD, while the American guidelines suggest evaluating multiple risk factors associated with worse prognosis [48]. In both cases, demographic (age), clinical (history of syncope, cardiac arrest, sustained ventricular tachycardia, and family history of SCD), and echocardiographic (maximum LV wall thickness, LA size, LVOT gradient, EF, and LV apical aneurysm) data are considered [47,48]. CMR for arrhythmic risk stratification is considered a second-line tool in both the American and European guidelines, particularly when the initial screening does not clearly identify a patient as high-risk or when the decision regarding ICD implantation remains uncertain [21,47,48]. Approximately 40% of patients with HCM progress to heart failure [42], with different clinical presentations linked to multiple underlying pathophysiological features, such as left ventricular out-flow obstruction, non-obstructive physiology with preserved EF, and end-stage systolic dysfunction. The main mechanisms involved appear to be myocardial ischemia, microvascular dysfunction, and replacement fibrosis, and CMR is key in detecting tissue abnormalities underneath [49]. Patchy, multiple stress-induced perfusion defects are frequent in perfusion CMR of HCM patients, reflecting microvascular disease. This finding was shown to be significantly associated with higher LGE volume and apical aneurysms in a recent study [50]. Indeed, multiple ischemic insults result in replacement fibrosis, which in turn leads to enhanced ventricular stiffness, diastolic disfunction, and, ultimately, LVEF impairment. Subsequently, LGE extent was found to reliably predict the development of heart failure in a population of 543 patients [51]. Moreover, LGE progression over time is also relevant, as it was significantly associated with LV dilatation, reduced ejection fraction, and GLS [52]. In another study, Yang et al. [53] conducted a retrospective, single-center study on 497 patients with hypertrophic cardiomyopathy to assess the prognostic value of LGE. Consistent with previous research, extensive LGE (≥15% of left ventricular mass) was associated with a higher risk of adverse outcomes. However, in patients with non-extensive LGE (<15% of LV mass) the subendocardial location of LGE, regardless of its extent, was a significant predictor of poor prognosis. The study suggested that both the extent and location of LGE (particularly subendocardial involvement) could refine risk stratification in HCM, especially for patients without extensive fibrosis (Figure 3). ECV is proven to be increased prior to LV hypertrophy development in sarcomere mutation carriers, serving as an early marker of disease progression [54]. In addition, LV strain assessment is effective in subclinical stages of the disease, as research demonstrated its ability to reliably predict elevated NT-proBNP and TnT values in a group of HCM patients with preserved EF [55].

Moreover, CMR is an accurate method for assessing wall thickness and other parameters relevant to arrhythmic risk stratification. Regarding the definition of wall thickness, the distribution of hypertrophy in HCM is heterogeneous and may be focal (limited to one to two segments, as seen in up to 10% of HCM patients), diffuse, or non-contiguous and can involve any myocardial segment [56]. The increasing use of CMR has prompted several studies evaluating the reliability of ventricular wall thickness measurements by TTE. These studies have demonstrated that CMR is superior to TTE in selected cases. Hypertrophy is commonly missed by TTE in patients with acoustic shadowing and poor endocardial definition, in patients with a focal involvement of anterolateral LV free wall or apex, and in the isolated involvement of the right ventricle [56]. In obstructive forms of HCM, CMR can also be useful in determining the indication for myectomy when echocardiographic assessment is inconclusive regarding the necessity of the procedure or the mechanism of obstruction [57].

### 3.5. Fabry Disease

**Figure 4:**
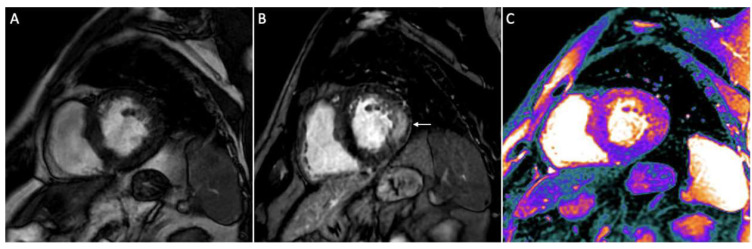
Short axis views in Fabry disease. Cine sequence showing left ventricular hypertrophy (**A**); typical LGE distribution in mid-wall area of infero-lateral segment (**B**; arrow); global reduction of native T1-mapping (purple areas; global native T1: 943 ms) except for infero-lateral wall (**C**).

Fabry disease is a hereditary condition arising from a deficiency of alpha-galactosidase, leading to globotriaosylceramide accumulation within lysosomes and subsequent clinical manifestations. The heart is one of the main affected organs, along with the kidneys and brain [11]. Although diagnosis is based on the results of an enzymatic activity test on a blood sample and a genetic test, CMR still plays a fundamental role in the assessment of these patients. Although there may be no apparent systolic dysfunction as EF is usually preserved, GLS is often impaired. A recent study demonstrated that GLS reduction has been observed prior to significant LV hypertrophy, suggesting its value as an early marker of cardiac involvement [58]. T1 mapping is the most informative CMR sequence in this setting. In Fabry disease, early T1 shortening reflects glycosphingolipid accumulation (Figure 4), which continues as LV hypertrophy develops. In advanced stages, T1 may pseudo-normalize due to interstitial fibrosis, a pattern that aids in differentiating Fabry disease from other causes of LV hypertrophy [59]. LGE appears in later disease stages, reflecting advanced fibrosis, typically in a midwall pattern of the inferolateral LV wall (Figure 4) [60]. When it is associated with normal values of T1, the suspicion of Fabry disease should be raised. Although LGE patterns and location are not specific for diagnosis, LGE still has a prognostic role as it has been found to be associated with adverse outcomes [61]. Moreover, the severity of LV wall thickening and hypertrophy correlates with an increased risk of ventricular arrhythmias [62]. Notably, the LV mass index assessed by CMR has demonstrated superior prognostic value for adverse cardiovascular outcomes compared to the LV mass index measured by 2D echocardiography [63]. Furthermore, CMR quantifies myocardial trabeculation and papillary muscle hypertrophy, contributing to LV mass assessment [64], with trabecular mass and complexity increasing in advanced disease stages [65]. Enzymatic replacement therapy (ERT) is the widely accepted etiological therapy for Fabry disease, as it halts its progression. Its early initiation is linked to better outcomes, since regression of both LV wall thickness and mass have been reported [66]. As the disease progresses toward its terminal stages and fibrosis develops, the efficacy of treatment is significantly reduced [67]. Therefore, the importance of CMR lies in its ability to raise the suspicion of Fabry disease, facilitating early diagnosis and treatment initiation.

### 3.6. Infiltrative Cardiomyopathies

**Figure 5:**
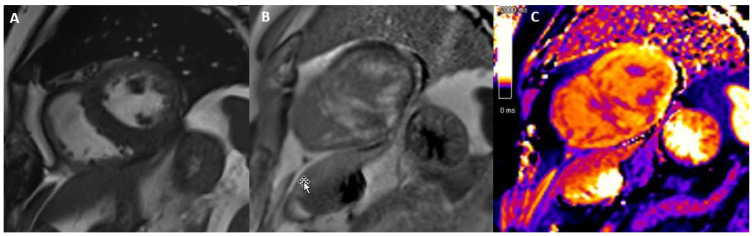
Transthyretin amyloidosis. Cine-imaging showing left ventricular hypertrophy (**A**). Diffuse patchy LGE (**B**). Very high T1 mapping and ECV values (1190 ms and 45%, respectively) (**C**).

Infiltrative cardiomyopathies encompass a wide range of both inherited and acquired conditions, which can result in a variety of clinical manifestations and ultimately lead to heart failure. Cardiac amyloidosis (CA) is characterized by the accumulation of misfolded proteins (amyloid fibrils) within the heart. The specific misfolded protein that is responsible for the development of the disease identifies different forms of CA, with light chain (AL) and transthyretin (ATTR) being the most prevalent [68]. Although rare, its prognosis is extremely poor if left untreated, hence its early detection is fundamental to improve outcomes. Although CMR is not sufficient itself to diagnose this condition, it is an essential part of the initial evaluation and ongoing management of these patients. The main morphological features of CA include biventricular hypertrophy with reduced end-diastolic volumes, reduced GLS with the typical apical sparing pattern, thickening and fibrosis of the heart valves, biatrial enlargement, sparkling texture of the myocardium, and pericardial effusion. None of these characteristics are pathognomonic, but their combination is quite specific and strongly indicative of amyloidosis [69]. CMR can provide further information through tissue characterization. Native T1 values are usually significantly elevated, reflecting amyloid deposition in the extracellular space (Figure 5). T1 mapping provides a quantitative measurement of T1 values, thus ensuring optimal diagnostic accuracy. Native T1 values tend to be higher in AL than in ATTR amyloidosis, likely due to the inflammatory response associated with light chain toxicity, a hypothesis further supported by elevated T2 values—indicative of inflammatory edema—in untreated AL patients [70,71,72]. Following the administration of gadolinium, post-contrast T1 mapping is performed to allow ECV estimation, that is, as expected, markedly elevated. Nevertheless, for the reasons outlined above, the elevation is greater in ATTR than in AL [73]. In advanced stages of the disease, the myocardium may retain more gadolinium than blood, making it harder to distinguish damaged tissue using standard imaging techniques. The phase-sensitive inversion recovery (PSIR) technique is crucial in this case, as it does not depend on operator settings. PSIR allows the normal myocardium to appear dark while affected tissue appears bright, providing clearer imaging. Both the presence and extent of LGE are linked to worse prognosis, while its distribution also reflects disease progression [74,75]. Initially, LGE appears in the subendocardium, particularly in the basal segment; as the disease advances, it becomes transmural [74]. T1 mapping and ECV trends are emerging markers of disease stage and treatment response, with ECV able to detect early cardiac amyloidosis even before LGE appears [76]. Several studies have demonstrated that the cardiac amyloid burden quantified by ECV is associated with adverse cardiac remodeling and increased all-cause mortality in patients with both AL and ATTR amyloidosis [77]. Also, a reduction in or absence of progression in ECV has been associated with treatment response in both ATTR and AL amyloidosis, demonstrating greater accuracy than other imaging parameters [78,79,80]. Besides tissue characterization, CMR allows for the assessment of various functional parameters that have prognostic implications in CA (including EF, GLS, and myocardial contraction fraction), with greater reproducibility than echocardiography [81].

### 3.7. Cardiac Sarcoidosis

**Figure 6:**
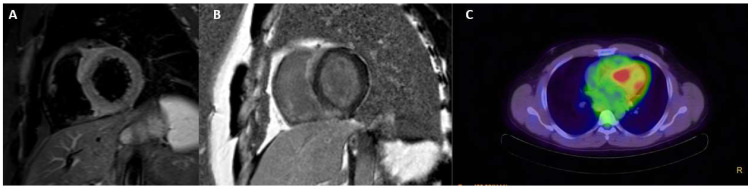
Cardiac sarcoidosis. CMR T2 imaging showing septal oedema (**A**). Septal LGE (**B**). Corresponding FDG-PET imaging showing high septal inflammation (**C**).

Sarcoidosis is a multisystem disorder of unknown etiology characterized by pathognomonic noncaseating epithelioid cell granulomas. Although the lungs and lymph nodes are the most frequently affected organs, the heart may also be involved and cardiac sarcoidosis (CS) is known to have a poor prognosis [82]. Therefore, an early diagnosis is crucial so that immunosuppressive therapies can be started without delay. A definitive diagnosis of CS requires histological confirmation. However, CMR can show characteristic features of CS and is therefore recommended as part of a multiparametric diagnostic pathway [83]. Regional wall motion abnormalities, ventricular wall thickening or thinning, atrial wall hypertrophy, increased echogenicity, and valve regurgitation due to papillary muscle involvement are some of the features seen in cine CMR sequences as a result of inflammation and granuloma formation within the myocardium. T1 and T2 mapping allows for direct measurement of tissue inflammation and has been found to be significantly elevated when compared to healthy controls. In CS, LGE marks granulomatous lesions and fibrotic areas (Figure 6). Due to the multifocal nature of the disease, LGE is typically patchy, so that no specific location or pattern may be identified [84,85]. CMR is a valuable tool for both diagnosis and risk stratification in cardiac sarcoidosis (CS). Several studies have correlated the presence and extent of LGE in sarcoidosis patients with poor prognosis and arrhythmic risk. Two metanalysis involving 694 and 760 subjects showed that positive LGE in CS patients was associated with higher odds for all-cause mortality, cardiovascular death, and ventricular arrhythmogenic events (ventricular arrhythmia, ICD shock, and sudden cardiac death) [86,87]. The presence of LGE was associated with poor prognosis even in patients with preserved LVEF, as shown in a study of 205 extracardiac sarcoidosis patients with LVEF > 50%, where a positive LGE on MRI was linked to a 20-fold higher annual risk of ventricular arrhythmias compared to LGE-negative patients [88]. Furthermore, even in cases of severely impaired LVEF, the absence of LGE was linked to a low risk of major cardiovascular events [87]. Based on this evidence, primary prevention ICD implantation is recommended in the presence of LGE on MRI after resolution of the acute inflammatory phase, regardless of LVEF [21].

### 3.8. Iron-Induced Cardiomyopathy

**Figure 7:**
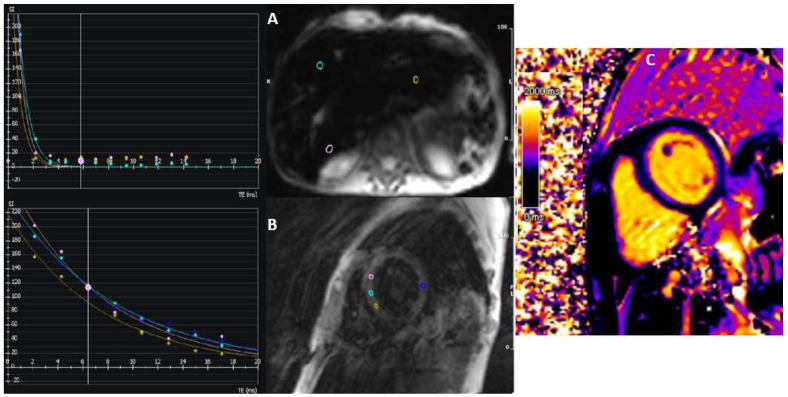
Graphs of the relaxation of T2* values over time to compute T2* of the liver (**A**) and myocardium (**B**). T2* values are consistent with severe iron overload of the liver (0.7 ms) and myocardium (8 ms). T1 mapping values are also very low (about 770 ms) (**C**).

Iron-induced cardiomyopathy is a direct consequence of myocardial iron overload. This can be due to genetic-inherited disease (e.g., hemocromatosis), but it is more prevalent in patients requiring systematic blood transfusion therapy (mainly because of talassemia major). This is a progressive disease, ultimately leading to heart failure and fatal arrhythmias. However, chelation therapy has greatly improved the prognosis of these patients [89,90]. CMR is the only technique currently available for the non-invasive quantification of myocardial iron burden. In this context, the most useful parameter is myocardial T2*, as it provides a direct measurement of iron overload. T2* is indicative of local magnetic field inhomogeneities, which increase with significant iron accumulation. Thus, T2* relaxation time declines in a direct and proportional manner with the rise in iron load [91]. Nowadays, a single mid-ventricular slice approach is the most widespread technique. However, a multislice approach, as demonstrated in a recent study, has been shown to facilitate the identification of segments that are more susceptible to iron deposits. It has been observed that anterior and inferior segments are the most frequently affected, while the inferior region appears to be involved only in the latter stages [92]. However, both approaches have been validated and can be useful in the early detection of the disease. Importantly, T2* can detect cardiac iron overload before echocardiographic abnormalities appear, as ventricular dysfunction typically manifests only after substantial iron accumulation (Figure 7) [93]. Early detection of cardiac iron deposition allows for timely initiation of iron chelation therapy, which can reverse iron overload and prevent cardiac dysfunction [94,95]. A study involving 652 patients with thalassemia demonstrated that T2* values < 10 ms were present in 98% of patients who developed heart failure and that the likelihood of left ventricular dysfunction increased progressively with decreasing T2* values [96]. Furthermore, T2 values < 20 ms were associated with a 4.6-fold increased risk of arrhythmias, with the risk increasing in stepwise fashion as T2* decreased [96]. Beyond diagnosis and risk stratification, T2* is also an effective tool for monitoring treatment response, particularly in assessing the efficacy of iron chelation therapy. Several randomized trials have demonstrated its utility in guiding treatment adjustments to improve patient outcomes [97,98].

### 3.9. Arrhythmogenic Cardiomyopathy

**Figure 8:**
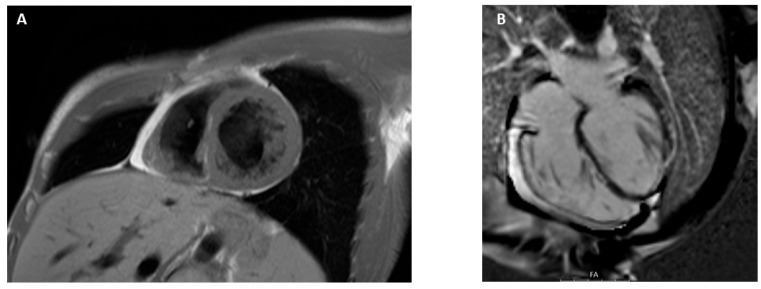
Arrhythmogenic cardiomyopathy. Short-axis view, black-blood flow, showing right ventricle and interventricular septum fatty infiltration (**A**). Four chamber view showing LGE in both ventricular chambers (**B**).

Fibro-fatty myocardial replacement is a hallmark of arrhythmogenic cardiomyopathy, a genetically determined heart muscle condition that is clinically linked to sudden cardiac death and malignant ventricular arrhythmias [99]. Three phenotypic variants have been identified: the classic right-dominant arrhythmogenic right ventricular cardiomyopathy (ARVC), a left-dominant form with predominant LV involvement, and a biventricular form affecting both ventricles [100,101]. Fibro-fatty myocardial replacement leads to functional myocardial changes (regional wall motion abnormalities, such as akinesia, dyskinesia, or dyssynchronous RV contraction) ultimately resulting in systolic dysfunction [100,101]. The severity of structural and functional anomalies involving RV can be graded using quantitative imaging reference values, as described by the 2010 International Task Force (ITF) criteria [102]. The 2020 International Expert Consensus highlighted limitations in diagnosing left-sided ACM variants, particularly the absence of specific criteria and CMR-based tissue characterization [103,104]. The recently introduced Padua criteria address these gaps by providing an updated diagnostic framework that emphasizes the central role of CMR across all ACM phenotypes, including biventricular and left-dominant forms [105]. Cine sequences for LV and RV, black blood flow, and LGE pictures should be included in the CMR study protocol for patients with suspected ACM. Fatty infiltration of the myocardium can be effectively detected using T1 (visible as an hyperintense signal relative to normal myocardium) and LGE sequences (Figure 8) [104]. T2-weighted images can detect myocardial edema and inflammation, which are hallmarks of myocarditis-mediated episodes of acute myocyte necrosis. These findings characterize the ‘hot phase’ of the disease, a rare clinical manifestation ACM that occurs more frequently in children and in individuals with desmoplakin gene mutations, associated with troponin release and chest discomfort [106,107]. The presence and extent of LGE in ACM are associated with a worse prognosis, including an increased risk of arrhythmic events and progression to heart failure [108]. Tandri et al. [109] examined 30 cases comparing contrast-enhanced CMR results with electrophysiological and endomyocardial biopsy results. The investigators came to the conclusion that there was a correlation between the inducibility of arrhythmias on electrophysiological investigation and the presence of RV LGE [109]. Also, the study showed that heart failure–related events were associated with the presence of left ventricular LGE and with elevated native T1, T2, and extracellular volume values [109]. In particular, the presence of LV LGE was linked to a worse prognosis compared to forms with isolated right ventricular involvement or without LGE [108]. Finally, as well as for T2, high values of native T1 and ECV are considered predictors of worse outcomes [110].

## 4. CMR Applications in Ischemic Heart Disease

CMR is a cost-effective, non-invasive imaging modality that provides comprehensive assessments of myocardial ischemia, viability, and cardiac function. It also plays a crucial role in risk stratification and guiding treatment strategies for patients with coronary artery disease (CAD). Several techniques are available for evaluating myocardial viability and predicting functional recovery post-revascularization, with an integrated approach using multiple CMR methods enhancing diagnostic accuracy. One method involves measuring segmental end-diastolic wall thickness (EDWT), which is a highly sensitive (95%) but less specific (41%) predictor of functional recovery. Myocardial thinning secondary to infarction correlates with infarct transmurality and reflects the extent of scar tissue. Notably, a myocardium with a wall thickness of less than 6 mm had a low probability of functional improvement following revascularization [99]. Another approach is dobutamine stress MRI, which has demonstrated good specificity (83%) but moderate sensitivity (74%), comparable to dobutamine echocardiography [111]. This technique evaluates myocardial contractile reserve and provides valuable insights into viability. Contrast-enhanced MRI offers a direct assessment of scar tissue extent, serving as a key tool in determining infarct severity, predicting post-revascularization functional recovery, and evaluating both short- and long-term outcomes [112]. Studies indicate that the probability of regional contractility improvement decreases as the transmural extent of LGE increases [113]. For example, in one study, 78% of dysfunctional myocardial segments without LGE showed contractile improvement after revascularization, whereas only 2% of segments with scar tissue extending beyond 75% of the LV wall exhibited functional recovery [114]. Moreover, infarct size is a critical determinant of adverse LV remodeling [115], and both the presence and extent of myocardial scarring were strongly and independently associated with major adverse cardiac events (MACE) and cardiac mortality [116].

CMR can also guide coronary revascularization by providing a functional assessment of coronary stenosis. Stress perfusion CMR has demonstrated high diagnostic accuracy, with a sensitivity of 89%, specificity of 87%, and excellent positive (91%) and negative (94%) predictive values for identifying hemodynamically significant CAD when compared to invasive fractional flow reserve (FFR) [117]. Regarding primary prevention ICD implantation, CMR serves as a non-invasive, accurate, and reproducible alternative to conventional echocardiography for evaluating ventricular volumes and function [118]. Regional ventricular function can be assessed both qualitatively (normal, hypokinetic, akinetic, or dyskinetic) and quantitatively (relative or absolute wall thickening and wall motion analysis), further enhancing its role in clinical decision making [119].

## 5. CMR Applications for Cardiac Resynchronization Therapy

Cardiac resynchronization therapy (CRT) has significantly improved the management of patients with symptomatic heart failure, severe LV dysfunction, and prolonged QRS duration. However, up to 30–45% of patients fail to experience clinical or echocardiographic benefits from CRT [120]. Enhancing response rates remains a key research focus, with evidence suggesting that targeting optimal LV pacing sites can improve outcomes [121,122]. The placement of the LV lead in or near a myocardial scar was associated with poorer outcomes [123,124], whereas placement in or near the region of latest mechanical contraction enhanced response rates and long-term prognosis [121,122]. However, standard fluoroscopic projections used during CRT implantation do not provide information on myocardial tissue characteristics, making it challenging to identify the optimal LV pacing site.

CMR has emerged as a promising tool for guiding LV lead placement by assessing myocardial scar distribution through LGE imaging and evaluating mechanical dyssynchrony using feature tracking [124]. Several studies have demonstrated the feasibility of real-time CMR-guided LV lead implantation, showing improved outcomes in patients who undergo this technique. These benefits include greater LV reverse remodeling, lower cardiac mortality, and fewer heart failure-related hospitalizations, highlighting the potential of CMR in optimizing CRT therapy [121,122,124].

## 6. Future Research and Perspectives

Future research in the application of CMR in heart failure is focused on enhancing diagnostic accuracy, refining patient stratification, and improving treatment outcomes. As heart failure remains a leading cause of morbidity and mortality, CMR offers a unique non-invasive approach to assess myocardial structure, function, and tissue characterization. Current parametric mapping sequences contain numerous dependent variables that might affect values, including sequence type, heart physiology, and others, and they require separate acquisitions for T1 and T2. A novel technique that allows for simultaneous and repeatable measurements of T1 and T2 mapping values in a single scan is CMR fingerprinting. Faster acquisition times, greater value consistency because there are no dependent variables, and elimination of the need for exogenous contrast agents are some possible advantages of this method. Future studies will likely explore the integration of CMR with other imaging modalities, such as echocardiography and molecular imaging, to enable a more comprehensive and personalized approach to managing heart failure. Moreover, ongoing efforts in developing artificial intelligence algorithms to analyze CMR data could significantly improve the speed and accuracy of diagnosis, facilitating early intervention and better patient outcomes.

## 7. Conclusions

In conclusion, CMR has emerged as a pivotal tool in the diagnosis, assessment, and management of heart failure. Its ability to provide detailed, non-invasive imaging of myocardial structure, function, and tissue composition has significantly enhanced our understanding of the disease. CMR techniques such as T1 and T2 mapping, LGE, and myocardial strain imaging offer valuable insights into the pathophysiology of heart failure, allowing for more accurate patient stratification and better-targeted therapies. Finally, CMR stands poised to play a central role in the future of heart failure management, contributing to more personalized and effective care for patients with this complex and debilitating condition.

## Data Availability

Not applicable.
